# ColoType: a forty gene signature for consensus molecular subtyping of colorectal cancer tumors using whole-genome assay or targeted RNA-sequencing

**DOI:** 10.1038/s41598-020-69083-y

**Published:** 2020-07-21

**Authors:** Steven A. Buechler, Melissa T. Stephens, Amanda B. Hummon, Katelyn Ludwig, Emily Cannon, Tonia C. Carter, Jeffrey Resnick, Yesim Gökmen-Polar, Sunil S. Badve

**Affiliations:** 1 Department of Applied and Computational Mathematics and Statistics, Harper Cancer Research Institute, University of Notre Dame, 102B Crowley Hall, Notre Dame, IN 46556 USA; 2 Genomics and Bioinformatics Core Facility, University of Notre Dame, Notre Dame, IN USA; 3 Department of Chemistry and Biochemistry, Comprehensive Cancer Center, The Ohio State University, Columbus, OH USA; 4 Functional Genetics Section, Genetics Branch, Center for Cancer Research, National Cancer Institute, Bethesda, MD USA; 5 Center for Precision Medicine Research, Marshfield Clinic, Marshfield, WI USA; 6 Department of Pathology, Marshfield Clinic, Marshfield, WI USA; 7 Department of Pathology and Laboratory Medicine, Indiana University School of Medicine, Indianapolis, IN USA; 8 Indiana University Melvin and Bren Simon Cancer Center, Indianapolis, IN USA

**Keywords:** Cancer genomics, Gastrointestinal cancer

## Abstract

Colorectal cancer (CRC) tumors can be partitioned into four biologically distinct consensus molecular subtypes (CMS1-4) using gene expression. Evidence is accumulating that tumors in different subtypes are likely to respond differently to treatments. However, to date, there is no clinical diagnostic test for CMS subtyping. In this study, we used novel methodology in a multi-cohort training domain (n = 1,214) to develop the ColoType scores and classifier to predict CMS1-4 based on expression of 40 genes. In three validation cohorts (n = 1,744, in total) representing three distinct gene-expression measurement technologies, ColoType predicted gold-standard CMS subtypes with accuracies 0.90, 0.91, 0.88, respectively. To accommodate for potential intratumoral heterogeneity and tumors of mixed subtypes, ColoType was designed to report continuous scores measuring the prevalence of each of CMS1–4 in a tumor, in addition to specifying the most prevalent subtype. For analysis of clinical specimens, ColoType was also implemented with targeted RNA-sequencing (Illumina AmpliSeq). In a series of formalin-fixed, paraffin-embedded CRC samples (n = 49), ColoType by targeted RNA-sequencing agreed with subtypes predicted by two independent methods with accuracies 0.92, 0.82, respectively. With further validation, ColoType by targeted RNA-sequencing, may enable clinical application of CMS subtyping with widely-available and cost-effective technology.

## Introduction

Colorectal cancer (CRC) is the fourth most common cancer in the U.S. and the second leading cause of cancer deaths. Attempts to reduce CRC deaths have been complicated by the molecular heterogeneity of the disease leading to differential responses to therapies. Over the past decade, numerous gene expression-based subtype systems were developed to better characterize CRC^1–6^. To resolve potential disparities between these systems, the Colorectal Cancer Subtyping Consortium (CRCSC) published the consensus molecular subtypes (CMS), which partitioned CRC tumors into CMS1, CMS2, CMS3, CMS4 and a small set of unclassified tumors^7^. Since publication of the CMS subtypes, numerous studies have explored how patients in different subtypes exhibit differential sensitivities to commonly used drugs^8–15^, contributing evidence to the clinical utility of CMS.

Progress towards clinical application of the CMS subtypes has been limited by the complexity of the CMS subtyping method. CRCSC created the CMSclassifier computer application for CMS subtyping using expression of hundreds of genes from whole-genome data^7^. The CMScaller application also uses whole-genome data and expression values of over 500 genes to report the CMS subtypes for tumor samples and preclinical models^16^. To analyze formalin-fixed, paraffin-embedded (FFPE) tissue samples collected in clinical studies, researchers have developed in-house classifiers using NanoString nCounter system^8,9,11,17^, microarrays^13^, or immunohistochemistry^18^. However, to date, there is no commercially available assay that meets the quality-control requirements of a clinical diagnostic test.

In this study, with the ultimate goal of producing a clinical assay for CMS subtyping, we developed the ColoType scores and classifier for CMS subtyping based on expression of 40 genes, implemented by whole-genome analysis or by targeted RNA-sequencing with an FFPE tissue source.

## Materials and methods

### Whole-genome data from publicly available patient cohorts

Genome-wide gene expression data from multiple cohorts generated using multiple platforms (two microarray platforms and RNA-sequencing) were collated for development and validation of ColoType.

*Cohort A* Cohort A (n = 1,888, Table 1) consisted of gene expression data from colon cancer samples hybridized to Affymetrix hgu133plus2 arrays comprised of the datasets GSE13067, GSE13294, GSE14333, GSE17536, GSE2109, GSE23878, GSE35896, GSE37892, GSE39582, KFSYCC. Duplicate samples in GSE14333 and GSE17536 were eliminated. Gene expression values normalized using fRMA^19^ were obtained from the Synapse Project 2623706 for all datasets except GSE2109. For GSE2109, the CEL files available on the Synapse archive were normalized using fRMA and batch-effect corrected to the other cohorts using ComBat^20^ from the SVA R package.

**Table 1:** Characteristics of colon cancer samples used for training and validation of ColoType.

	Cohort A (Affymetrix)		Cohort B (PETACC-3)		Cohort C (COAD)	
	Training (n = 683)^a^	Validation (n = 1,205)^b^	Training (n = 342)	Validation (n = 346)	Training (n = 189)	Validation (n = 193)
Assay platform	Affymetrix hgu133plus2	Affymetrix hgu133plus2	custom array	custom array	RNA-seq	RNA-seq
**Stage**						
1	61	134	NA	NA	28	29
2	293	206	NA	NA	65	62
3	241	164	NA	NA	58	48
4	88	30	NA	NA	15	14
NA	0	612	NA	NA	7	2
Median age	68	70	NA	NA	67	69
**Microsatellite instability**						
MSI	72	86	NA	NA	32	28
MSS	402	133	NA	NA	133	123
NA	209	653	NA	NA	8	4
**CMS**^c^						
CMS1	117	135	39	39	28	26
CMS2	281	315	120	121	60	50
CMS3	81	121	36	37	25	24
CMS4	141	200	72	73	41	37
NONE	63	101	75	76	19	18

^a^Formed from datasets (n): GSE17536 (167), GSE39582 (516).

^b^Formed from datasets (n): GSE13067 (69), GSE13294 (150), GSE14333 (149), GSE2109 (264), GSE23878 (31), GSE35896 (57), GSE37892 (121), KFSYCC (276).

^c^The CMS classification as reported by CRCSC combining the network and random forest classifiers, termed CMS-final in this study.

Clinical characteristics and the Colorectal Cancer Subtyping Consortium (CRCSC) consensus molecular subtype classifications for each sample in Cohort A were also obtained from Synapse Project 2623706, and summarized herein (Table 1). For the purpose of deriving and validating the ColoType CMS scores, Cohort A was partitioned into training (n = 683) and validation (n = 1,205) sets. The training set was defined to be all samples in the datasets GSE17536 and GSE39582. The CRCSC reported^7^ CMS classifications obtained by network analysis, their random forest classifier, and a classification we term “CMS-final” that agreed with the network classification when that process produced a result, and agreed with the random forest classifier for samples unclassified by the network method. In this paper the term “CRCSC CMS subtype” refers to “CMS-final” unless noted otherwise.

*Cohort B* Cohort B (n = 688) consisted of samples from the PETACC-3 clinical trial^21^ for which mRNA was hybridized to an Almac custom Affymetrix microarray platform. Gene expression and clinical data were obtained from Synapse Project 2623706. The cohort was randomly divided into training (n = 342) and validation (n = 346) sets balanced for CRCSC CMS subtype (Table 1).

Cohort C: Cohort C was formed from TCGA-COAD (n = 382)^22^. Raw RNA-sequencing (RNA-seq) data (BAM files) were obtained from Genomic Data Commons (https://gdc.cancer.gov). Following alignment to the Ensembl v90 genome build, gene expression was measured as the log base 2 of transcripts per million (TPM) derived from count values using the feature counts algorithm^23,24^. This realignment enabled better comparison between COAD expression data and whole-genome and targeted RNA-seq data for the Marshfield cohort. Patient-level clinical data and CMS classifications for COAD were obtained from Synapse Project 2623706. To support the development of ColoType, COAD was randomly divided into training (n = 189) and validation sets (n = 193) balanced for CRCSC CMS subtypes (Table 1).

To enable coordinated derivation in these multiple cohorts we restricted expression data to array features annotated to a unique ENTREZID, and further restricted to ENTREZIDs assayed in all three cohorts. For each such ENTREZID, we restricted expression to the array feature with maximal interquartile range in the cohort’s training set, among all features representing the ENTREZID. In the end, for a set of ENTREZIDs assayed in all three cohorts, there was a unique expression value for each sample.

### Size factor normalization of RNA-seq data

To enable comparisons between samples, raw RNA-seq data are typically normalized by dividing read counts by a sample-specific normalization factor. In whole-genome normalization methods, such as RPKM or TPM, the normalization factor involves the total reads for the sample and the gene length, inappropriate for targeted RNA-sequencing. For targeted RNA-sequencing, data from a reference sample can be used^25^. To enable more general application, we used a so-called pseudo-reference-sample scaling factor^26^, as applied in the DESeq R package.

Given a set **P** of samples, and raw counts for each gene in a set *G* and sample in **P**, expression values by size factor normalization are computed as follows.

- The *size factor* for gene *i* is the geometric mean of the raw counts for gene *i* for all samples in **P**;
- The *scaled count value* for a gene *i* and sample *j* is the raw count number divided by the size factor for gene *i*;
- The *size factor* for sample *j* is the median of the scaled counts in sample *j* over all genes in *G*;
- The *size factor normalized* expression value for gene *i* in sample *j* is then the scaled counts for gene *i* divided by the size factor for sample *j*.

Size factor normalization was applied to whole-genome sequencing data as well as targeted RNA-seq data to enable comparison of subtyping methods in data obtained by multiple sequencing methods. Modeling with size factor normalized data in this study used log2 transformed size factor normalized expression values as gene expression values.

### Marshfield cohort (novel)

Data on a cohort of stage 2 colon cancer patients was obtained from Marshfield Clinic, Marshfield, Wisconsin (Table 2). Clinical data from electronic health records, including follow-up for at least five years, and glass slides from formalin-fixed paraffin embedded surgical resection samples were obtained for each patient. Regions of tumor tissue were identified by pathologist review of H&E slides.

**Table 2:** Summary clinico-pathological features of the Marshfield cohort.

feature	Marshfield cohort (n = 49)
Age at surgery (median)	76.4
Stage 2	49
**T-stage**	
T2	1
T3	44
T4	4
**Location**	
Ascending	17
Transverse	21
Descending	2
Sigmoid	9
Distant metastases	7
Deaths	18
Median follow-up days	2,212

### RNA-seq analysis of Marshfield cohort

Tumor tissue was obtained from tumor-rich regions of unstained slides (1 or 2 per patient) and deparaffinized by Xylene wash. mRNA was then extracted with RecoverAll Total Nucleic Acid Isolation Kit.

FFPE RNA was prepared for sequencing using two independent library construction methods: RNA-seq using ribosomal RNA depletion, and AmpliSeq for Illumina Custom RNA targeted panel. RNA was quantitated by Qubit using the RNA HS Assay (Thermo Fisher Scientific Inc.) and assessed for integrity on the Bioanalyzer 2100 (Agilent Technologies Inc.). 80–100 ng of RNA was used as input for the library construction for both approaches. Quantifiable mRNA was obtained for 49 samples.

For whole-genome analysis, libraries were constructed using the NEBNext Ultra II Directional Library Prep kit with NEBNext rRNA Depletion module (New England Biolabs) (Cat. #E7765S, E6350L) following manufacturers recommendations. Libraries were quality assessed on the Bioanalyzer DNA 7,500 chip and quantified by qPCR using the Illumina Kapa Library Quantification Kit (Roche Diagnostics). The libraries were normalized, pooled in equal molar amounts, and sequenced on 6 lanes of a NextSeq v2.5 High Output flowcell using paired 75 bp reads. All libraries generated > 27 million raw reads. FASTQ files were aligned to the Ensembl v90 genome build, and gene counts computed using the feature counts algorithm^23,24^, and gene expression values computed by size factor normalization, log2 transformed.

ColoType panel genes served as targets for a custom AmpliSeq RNA panel. Libraries were constructed using the AmpliSeq for Illumina custom RNA panel reference guide (Illumina Inc.) following the standard workflow. Libraries were quality assessed on the Bioanalyzer DNA 7,500 chip and quantified by qPCR using the Illumina Kapa Library Quantification Kit. The libraries were normalized, pooled in equal molar amounts, and sequenced on 1 lane of a MiSeq v2 (300 cycle) kit using paired 150 bp dual indexed reads for an average output of 203,000 raw reads per library. FASTQ files were aligned to the Ensembl v90 genome build and gene counts computed using the feature counts algorithm^23,24^ and gene expression values computed as log2 of size factor normalized values, as for whole-genome sequencing data.

### Multigene risk score classification method

The cornerstone of a multigene expression-based clinical diagnostic test is the precise and reproducible quantification of species of mRNA. The multigene risk score (MRS) classification method was designed to classify samples by making efficient use of a small number of precisely measured mRNA species, perhaps including use of a reference set of data. Gene expression values are applied in an MRS classification system using a so-called risk score model of the gene, introduced in the EarlyR prognostic signature^27^. This topic is discussed below and in greater detail in Supplementary Methods S1.

#### Gene risk scores for classification

A risk score for a gene *g* in a patient cohort **P** is defined from the distribution of the gene’s expression values in **P** using the statistical method of mixture modeling^28–30^. In its simplest form, a mixture model of a gene with bimodal expression in **P** partitions the expression values into the two modes, a high component and a low component. In this case, a risk score for *g* would be near 0 for most samples in one mode, say the low component, and near 1 for most samples in the high component, and rising steeply from 0 to 1 near the threshold between the low and high components. A risk score is created using bootstrap resampling and differs from a binary variable by representing potential uncertainty in the value of the threshold. This methodology can also be applied to genes that are not bimodal but whose expression values have a somewhat irregular distribution, presenting several possible boundary points between high and low components. In this case, a risk score model of the gene could be defined around any boundary between the possible high and low components. Depending on the desired application, a risk score may be alternatively defined to be near 1 for the low component instead of the high component. In either case, the graph of a risk score is S-shaped or the mirror image of such.

In this study, risk scores were used to build scores predictive for subtypes. For a well-defined subtype *X* in a cohort **P**, there are typically genes that are significantly differentially expressed between the samples in and out of *X*. A risk score of a differentially expressed gene is a representation of the probability that a sample is in *X* based on the gene’s expression values. As such, a risk score is a single-gene predictive score for *X*.

For cohorts of patients **P** and **Q** that contain similar patient populations, the distributions of the expression values of a gene *g* in **P** and **Q** would be comparable. Similarly, we define risk scores in **P** and **Q** to be *equivalent* if they are models of the same gene and have comparable distributions (detailed in Supplementary Methods S1.1).

#### Multigene risk score (MRS) score to measure the probability of membership in a target subtype

We first identify genes *g*_1_,…,*g*_*n*_ whose risk scores *r*_1_,…,*r*_*n*_ are individually predictive of membership in a subtype *X* of a cohort **P**. The consensus prediction of membership in *X* by these risk scores can be represented initially by *r* = the mean of the risk scores; i.e., for a sample *v* in **P**, the value of *r* is the mean of the values of *r*_1_,…,*r*_*n*_ at *v*. The MRS score, defined as follows, better represents the probability that a sample is in the subtype. Assuming that **P** is a training set in which membership in the subtype *X* is known, define *p* = *MRS score* derived from *r*_1_,…,*r*_*n*_ in **P** so that for a sample *v* in **P**, the value of *p* at *v* is the positive predictive value of *r* at *v*; i.e., for *V* the samples in **P** with *r* value at least *r*(*v*), *p*(*v*) is the number of samples in both *X* and *V,* divided by the number of samples in *V*. Using a mathematical smoothing method, we can require that the MRS score is non-decreasing and a smooth curve.

An MRS score can be extended to other cohorts, e.g., a validation set, using data from the training set as a reference set of lookup tables. For example, to find the value of a risk score for a gene *g* and new sample *x*, we find the sample *y* in the training set whose expression value of *g* is closest to that of *x*, and define the risk score value of *x* to be that of *y* in the training set. The MRS score value for a new sample can be defined from means of risk score values by the same method.

#### Assignment of subtypes using a set of MRS scores

In this study, we defined an MRS score for each of the CMS subtypes. Using these, we classified a sample into the subtype whose MRS score value was above a classification threshold (Supplementary S1.2) and maximal among such scores. If no score of the sample was above the score’s classification threshold, the sample was unclassified.

### CMSclassifier, CMScaller and CRCassigner subtyping methods

Multiple subtyping systems were generated for comparison with ColoType. The CMSclassifier R package^7^, obtained from Synapse Project 2623706, was applied with the SSP (single sample predictor) method. The CMScaller R package^16^ (https://github.com/peterawe/CMScaller) was applied with the CMS template. CRCassigner subtyping was accomplished by applying the partitioning around medoids (PAM) methodology to the published table of 786 gene centroids^6^.

### Statistical methods

All statistical analyses were performed using R (https://www.r-project.org) version 3.6.2 and Bioconductor packages (https://bioconductor.org). The package *mclust*^28,29^ was used for fitting mixture models.

The ability of a continuous score to predict membership in a subtype was assessed with the receiver operator characteristic (ROC) curve which plots sensitivity (y-axis) by 1—specificity (x-axis) for all values of the score. The ROC curve is a test of how the accuracy of classification varies with values of the continuous score. The area under the ROC curve (AUC) gives a numerical measure of a score’s predictive significance. The quality of the prediction is better than random if AUC is > 0.5 and improves as AUC increases up to 1. The *plotROC* R package was used for these computations^31^.

ColoType defined a discrete classification that predicted the four subtypes of the CRCSC CMS classification. The quality of the prediction was assessed using the overall accuracy, along with the sensitivity, specificity and Cohen’s Kappa statistic. These statistics were computed using the confusionMatrix function of the *caret* R package^32^.

### Ethical approval and informed consent

The Marshfield cohort was created with the approval of the Marshfield Clinic Institutional Review Board (protocol number CAR60214). The IRB approved a waiver of informed consent because the research presented no more than minimal risk to study participants and the waiver of informed consent would not adversely affect the rights or welfare of participants. The protocol for this project was also approved by the IRB committees of the University of Notre Dame and Indiana University School of Medicine. All research was performed in accordance with relevant guidelines and regulations.

## Results

### Discovery of the continuous ColoType CMS subtyping scores

For each of CRCSC CMS1–4 we developed a continuous score that predicted membership in the subtype following the MRS score methodology (“Methods”). Here, we describe the process for CMS1; the process was identical for the other subtypes. The first step was to identify the panel of genes and their risk scores from which the predictive score was derived. To help reduce the potential bias due to a particular assay technology or tissue source we evaluated a gene’s predictive significance simultaneously in the training sets of Cohort A, B, and C, as they represent distinct analytical platforms. This was organized using triplets of risk scores (r_A_, r_B_, r_C_) so that each member of the triplet was a risk score of the same gene in Cohort A, B, C training sets, respectively, and the three risk scores were pairwise equivalent (“Methods”). We ranked all such triplets (representing 7,522 genes) by a consensus measure of significance in the three training cohorts as follows.

For each risk score in the Cohort A training set that was a member of such a triplet, we used a linear model to evaluate the significance of the risk score as a predictor of CRCSC CMS1 membership (using CMS-final as covariate and the risk score as response variable), and ranked all such risk scores in Cohort A training set by p-values of the linear models. We also ranked the other risk scores in the triplets by significance in the training sets of Cohort B and Cohort C, respectively. Finally, for each triplet, we computed the mean of the ranks of the three component risk scores, and ranked all of the triplets by these mean values, lowest to highest. This resulted in a ranked list of genes and triplets of the genes’ risk scores in the three training sets.

Candidates for the score to predict CMS1 in each training set were generated by MRS methodology using varying numbers of genes and evaluated by ROC analysis. Specifically, from the highest ranked 5, 7, 10, 15 and 20 triplets, and the risk scores for Cohort A training set from these triplets, we generated MRS scores for Cohort A training sets. The AUC values in Cohort A training set for MRS scores generated by 5, 7, 10, 15 and 20 risk scores were 0.95, 0.96, 0.98, 0.98, 0.98, respectively. We similarly generated scores for Cohort B and Cohort C training sets from the same highest ranked 5, 7, 10, 15 and 20 triplets. The AUC values of the corresponding scores in the Cohort B training set were 0.88, 0.89, 0.91, 0.95, 0.97, and for the Cohort C training set they were 0.97, 0.97, 0.98, 0.98, 0.98. Inspecting these results, we selected the 10-gene panel as the smallest one with the AUC values above 0.90 in the three cohorts.

The above method was repeated to identify optimal scores for CMS2, CMS3 and CMS4, all of which used 10 genes. The resulting score for CMS1 was defined to be the *ColoType CMS1-score*, similarly, for the other subtypes. See Supplementary Table S1 for a list of the 40 genes.

### Validation of the continuous ColoType CMS scores

**Figure 1:**
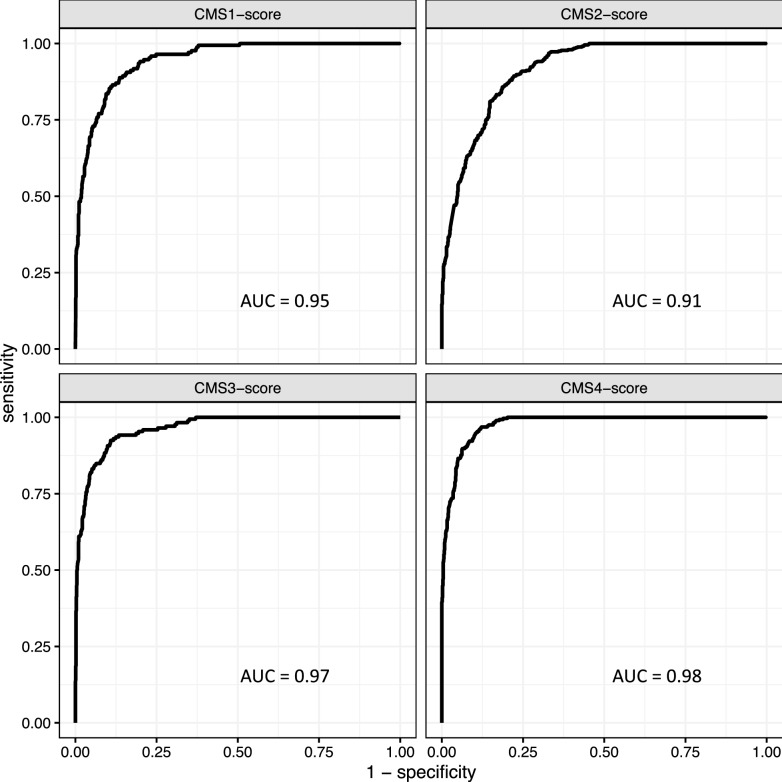
Receiver operator characteristic (ROC) curves are plotted for ColoType CMS1-score, CMS2-score, CMS3-score, and CMS4-score, for samples in the Cohort A validation set (n = 1,205). Area under the curve (AUC) values are displayed on the panels.

The ColoType CMS1-score was extended to all samples in Cohort A using the score in the Cohort A training set as a reference set (“Methods”), and similarly for other subtypes and cohorts. The significance of the scores as predictors of the CRCSC CMS subtypes in the validation sets were assessed with ROC plots and AUC values (Fig. 1 for Cohort A validation set, Supplementary Fig. S1 for Cohort B validation set and Supplementary Fig. S2 for Cohort C validation set). As the figures show, the AUC values for all subtypes and cohort validation sets were greater than or equal to 0.91.

### Assignment of the discrete ColoType CMS subtypes

Assignment of CMS subtypes using the ColoType scores followed MRS methodology (Supplementary Methods S1.5). The first step was to identify a classification threshold for each ColoType CMS score and each cohort as follows. A threshold of a continuous score predicting a subtype gives rise to a binary predictor by separating samples above or below the threshold. This associates to any potential threshold of the score a sensitivity, specificity and Youden index (sensitivity + specificity − 1) of subtype prediction. For CMS1 in Cohort A, e.g., we selected as the *classification threshold* the value of the CMS1-score that maximized the Youden index in the Cohort A training set among possible thresholds of the score value^33^. Classification thresholds were identified separately for each CMS score and each cohort (Table 3.)

**Table 3:** Classification thresholds for ColoType CMS scores in each cohort.

	CMS1-score	CMS2-score	CMS3-score	CMS4-score
Cohort A	0.751	0.792	0.663	0.684
Cohort B	0.450	0.760	0.447	0.612
Cohort C, log2 TPM normalized	0.790	0.698	0.564	0.767
Cohort C, size-factor normalized	0.630	0.613	0.632	0.613
Marshfield, whole-genome^a^	0.630	0.613	0.632	0.613
Marshfield, AmpliSeq^a^	0.630	0.613	0.632	0.613

^a^Classification thresholds for these cohorts were selected in reference to Cohort C with size-factor normalized gene expression.

Following identification of the classification thresholds, a sample in Cohort A was classified into the CMS subtype whose associated score was above the classification threshold and maximal among such scores; if no score was above the classification threshold the sample was unclassified. Samples in Cohort B and Cohort C were similarly classified using the corresponding ColoType scores and classification thresholds (Table 3).

### Measurement of the agreement between ColoType CMS subtypes and CRCSC subtypes

The degree of agreement between ColoType CMS subtypes and CRCSC subtypes in the combined validation sets from Cohorts A, B and C was assessed by confusion matrix (Table 4, n = 1,744, accuracy 0.90 95% CI 0.88–0.92, Kappa = 0.86). The samples unclassified by ColoType (16%) or the CRCSC classifier (14%) were excluded from calculation of the statistics. The levels of significance of the cohort-specific predictions of CRCSC subtypes by ColoType subtypes were: accuracy 0.90 95% CI 0.88–0.92, Kappa = 0.86, for Cohort A validation set, accuracy 0.91 95% CI 0.87–0.95, Kappa = 0.87 in Cohort B validation set, and 0.88 95% CI 0.82–0.93, Kappa = 0.83 in Cohort C validation set. ColoType scores and classifications for all samples in Cohorts A, B, C, along with the classification thresholds, are reported in Supplementary Table S2.

**Table 4:** Confusion matrix of ColoType CMS predictions and CRCSC CMS subtypes (CMS-final) in the combined validation sets.

ColoType	CRCSC subtypes (CMS-final)				
	CMS1	CMS2	CMS3	CMS4	NONE
CMS1	190	1	18	19	28
CMS2	0	529	4	59	65
CMS3	6	5	183	2	25
CMS4	13	4	0	290	31
NONE	33	88	31	32	88
**Statistics**^a^					
Sensitivity	0.91	0.98	0.89	0.78	
Specificity	0.99	0.92	0.99	0.98	

^a^Statistics are reported for prediction of each CMS subtype individually; unclassified samples were excluded from the computation.

### Comparison of multiple CMS subtyping methods using size factor normalized data in Cohort C

Subsequent assignment of CMS subtypes for the Marshfield cohort used size factor normalized gene expression data. We first used Cohort C to test for possible distortion of subtyping methods due to using size factor normalized data. Subtypes were generated using CMSclassifier and CMScaller in Cohort C with log2 size factor normalized expression values as described in Methods (Supplementary Fig. S3). The resulting classifications agreed with CMS-final with accuracies 0.90 95% CI 0.86–0.93 (CMSclassifier) and 0.91 95% CI 0.87–0.94 (CMScaller) and Kappa 0.86 (CMSclassifier) and 0.87 (CMScaller). We also computed ColoType for Cohort C with size factor normalized data as follows. We identified risk scores for panel genes equivalent to those used above, which were based on log2 TPM expression values. We then derived scores, classification thresholds (Table 3) and subtypes based on size factor normalized data in Cohort C, as done above with log2 TPM expression values. The resulting subtypes agreed with CMS-final with accuracy 0.89 95% CI 0.85–0.92 and Kappa = 0.85. These multiple classifications showed widespread agreement with CRCSC subtypes (Supplementary Fig. S3), verifying that size factor normalization did not bias these classifiers.

### Assignment of CMS subtypes for Marshfield cohort using multiple independent methods

**Figure 2:**
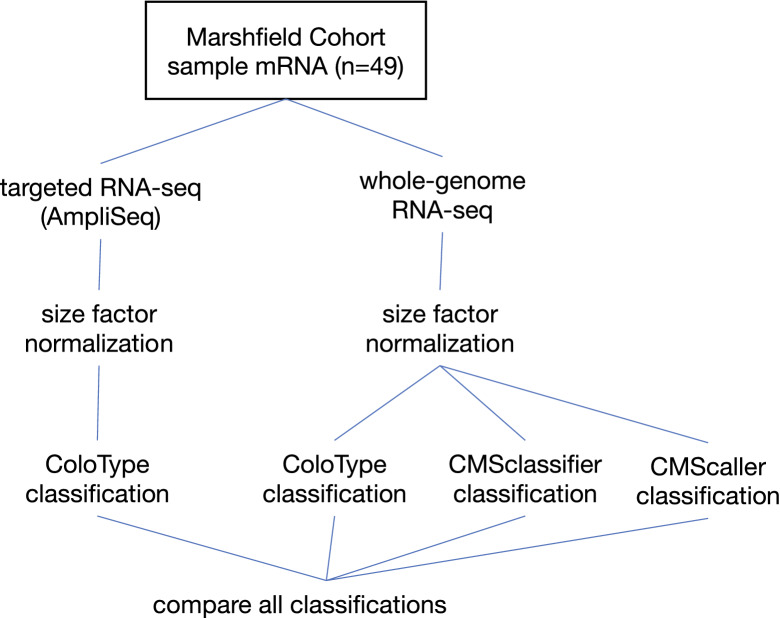
Multiple independent analyses were performed on the Marshfield cohort samples to assess the accuracy of subtyping by ColoType targeted RNA-seq analysis. Count data were generated by whole genome RNA-seq and by targeted RNA-seq with the ColoType custom AmpliSeq library. Gene expression values were computed from both sets of count data by size factor normalization, log2 transformed. ColoType was applied to expression data by AmpliSeq, and three independent classifiers were applied to whole-genome data. Results of the four subtype systems were compared.

We generated CMS subtypes for samples in the Marshfield cohort by multiple additional independent methods to assess the efficacy of the ColoType targeted RNA-seq assay (Fig. 2). To this end, we generated whole-genome transcription data from mRNA extracted from Marshfield cohort FFPE samples using RNA-seq with an rRNA depletion library preparation (“Methods”). To best enable comparisons with classifications using targeted RNA-sequencing, gene expression values were computed with size factor normalization (“Methods”).

Classifications by CMSclassifier (SSP method) and CMScaller were generated from the log2 size factor normalized expression values in the Marshfield cohort (“Methods”). For further comparison of subtyping methods, we also computed ColoType subtypes for Marshfield cohort with these expression values using Cohort C with size factor normalized expression values as a reference set. Specifically, for each panel gene we identified a risk score in the Marshfield cohort equivalent to the risk score of the gene in Cohort C. Then, for CMS1, we computed the mean of the risk scores of the 10 panel genes, for each sample, and then obtained a ColoType CMS1-score value for each sample by reference to the ColoType CMS1-score in Cohort C (“Methods”). Continuous scores for other subtypes were computed likewise. ColoType subtype classifications were computed using these continuous score values and the same classification thresholds as for ColoType in Cohort C with size factor normalized expression data (Table 3.)

Preservation of samples with formalin can degrade some species of mRNA, leading to degenerate expression values. Of note, expression of each of the 40 ColoType panel genes measured by whole-genome RNA-seq in the Marshfield cohort had distributions comparable to those in Cohort C. As a consequence, these 40 genes are appropriate candidates for an implementation of ColoType by targeted RNA-seq for FFPE samples.

### ColoType targeted RNA-seq assay: development and application to the Marshfield cohort

A custom library containing amplicons for the 40 ColoType panel genes was designed with AmpliSeq for Illumina custom RNA panel reference guide (Illumina Inc.) following the standard workflow.

For each sample in the Marshfield cohort, count values were then obtained for the 40 ColoType panel genes by AmpliSeq (“Methods”, Fig. 2), and gene expression values were computed with size factor normalization, log2 transformed. From these panel gene expression values, ColoType CMS scores and subtypes were computed using, as a reference set, Cohort C with size factor normalized expression values, as above for whole-genome sequencing data.

### Comparison of ColoType targeted RNA-seq subtypes with independent classifications

**Figure 3:**
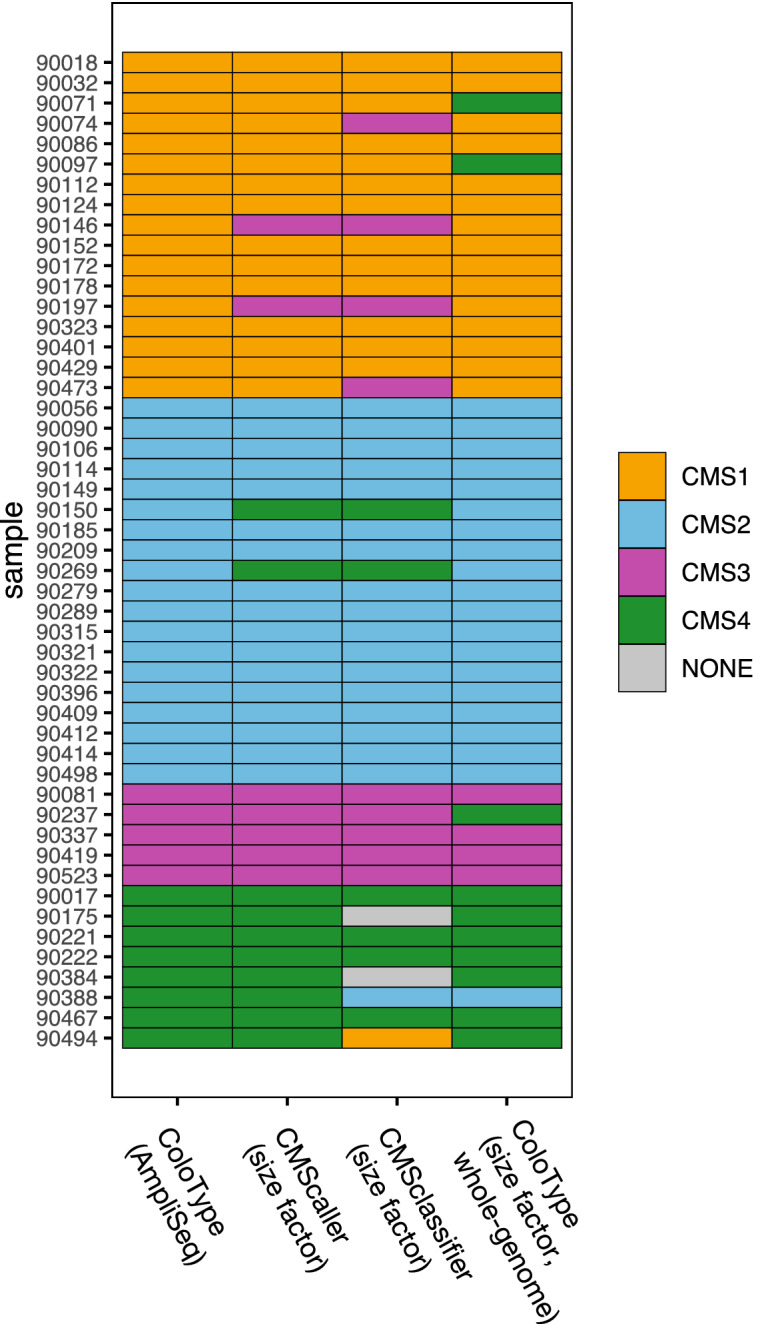
For each sample in the Marshfield cohort, the CMS classification is displayed for ColoType by targeted RNA-sequencing (AmpliSeq), and the classification methods CMScaller, CMSclassifier and ColoType by whole-genome data with size factor normalized expression values.

Comparisons of CMS subtypes by ColoType targeted RNA-seq analysis with those generated with whole-genome data showed high degrees of agreement (Fig. 3.) Specifically, ColoType targeted RNA-seq assay predicted CMScaller CMS classes with accuracy 0.92 95% CI 0.80–0.98, CMSclassifier with accuracy 0.82 95% CI 0.68–0.91, and ColoType whole-genome assay with accuracy 0.92 95% CI 0.80–0.98.

### Prediction of ColoType scores and subtype for a single new sample using a reference set

To be suitable for clinical use, a diagnostic test must be able to report results for a single new sample. For ColoType, a single-sample classifier can be defined in the context of any normalized gene expression measurement technology using a corresponding large reference set of samples (Supplementary Methods S1.5). For example, assume we have established a large reference set R of samples in which size-factor normalized expression values have been obtained for all ColoType panel genes using the AmpliSeq targeted RNA-seq library. Also assume that ColoType CMS score values and all the underlying risk scores have been computed for samples in R. Given a new sample s, the ColoType scores and subtype for s could be computed, as follows.

1. Obtain for s the size-factor normalized expression values for ColoType panel genes using the AmpliSeq targeted RNA-seq library;
2. For each panel gene g, identify a sample y in R whose expression value for g is closest to that of s; then associate to s the value of g’s risk score for y;
3. For each CMS subtype, and the 10 ColoType panel genes representing this subtype, compute the mean of the corresponding risk score values for s that were obtained in step 2;
4. For each CMS subtype, e.g., CMS1, identify a sample z in R whose mean risk score value is closest to that obtained for s in step 3; then associate to s the CMS1-score value of z;
5. Compute the ColoType CMS subtype of s from the CMS score values using the classification thresholds established for R.

### Prediction of CRCassigner subtypes using ColoType scores and subtypes

One of the CRC subtyping systems that contributed to the definition of CMS was CRCassigner^6^. CRCassigner partitions tumors into the Inflammatory, Enterocyte, TA, Goblet-like and Stem-like subtypes. Because this system has received significant further development^34^, we studied the ability to represent CRCassigner subtypes with ColoType scores and subtypes, beginning in Cohort A training set.

Using the published PAM algorithm (“Methods”), we computed the CRCassigner subtypes for Cohort A and compared these classifications with ColoType subtypes in the Cohort A training set (Supplementary Table S3). We found that TA was a subset of CMS2, and Enterocyte had large overlap with CMS2 but almost 50% of the subtype’s samples were in other CMS subtypes. Predominantly, CMS4 consisted of Stem-like and Enterocyte samples, CMS3 consisted of Goblet-like and Enterocyte, and CMS1 samples were largely Inflammatory, with a minority of Goblet-like and Enterocyte samples. Moreover, the ColoType scores predicted CRCassigner subtypes in the Cohort A training set with the following AUC values: 0.94 for CMS1-score prediction of Inflammatory, 0.90 for CMS2-score prediction of TA, 0.53 for CMS2-score prediction of Enterocyte, 0.93 for CMS3-score prediction of Goblet-like, 0.93 for CMS4-score prediction of Stem-like. Guided by these relationships, we developed a system for prediction of CRCassigner subtypes using ColoType scores and a single additional score to predict the Enterocyte subtype.

**Figure 4:**
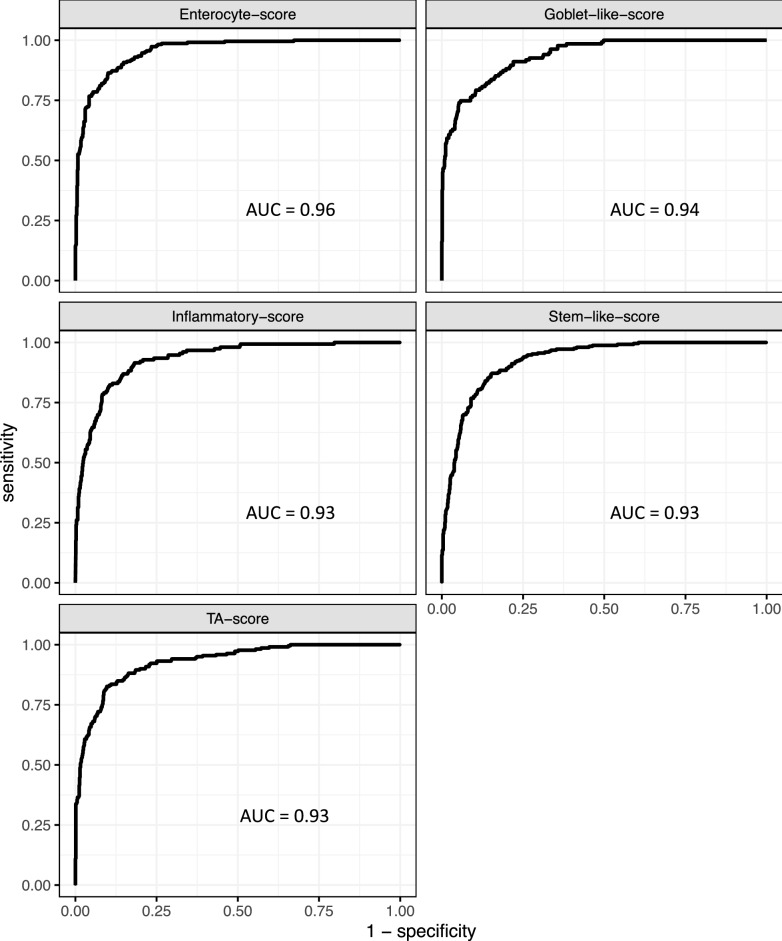
ROC curves are plotted in the Cohort A validation set (n = 1,205) for the scores developed herein to predict CRCassigner subtypes. The Enterocyte-score was derived from risk scores for genes *CA1* and *CA2*. The other scores were derived from the ColoType CMS scores and the Enterocyte-score.

Using the MRS score methodology in the Cohort A training set, we derived a continuous score (*Enterocyte-score*) to predict the Enterocyte subtype using the two genes *CA1* and *CA2* (AUC = 0.96 in Cohort A training set). Turning to other subtypes, recall that Stem-like was predicted by CMS4-score alone with AUC 0.93 in Cohort A training set. However, 17% of CMS4 in this cohort were Enterocyte samples. To better isolate Stem-like, we defined *Stem-like-score* to be CMS4-score minus Enterocyte-score; i.e., the score for sample s is the CMS4-score(s)–Enterocyte-score(s). This Stem-like-score predicted the Stem-like subtype with AUC 0.94 in Cohort A training. TA was approximated by CMS2-score, however the CMS2 subtype in Cohort A training also included significant numbers of Enterocyte and Stem-like samples. Accordingly, we defined the *TA-score* to be CMS2-score minus Enterocyte-score minus CMS4-score, which predicted TA in Cohort A training with AUC 0.94. We defined *Inflammatory-score* to be CMS1-score and *Goblet-like-score* to be CMS3-score because altering with other scores did not increase the AUC values. The predictive significance of these five CRCassigner subtype scores were assessed in Cohort A validation set by ROC analysis (Fig. 4); all scores reported AUC values at least 0.93.

A discrete classification algorithm based on the ColoType CMS subtypes, Enterocyte-score and CMS4-score was derived in Cohort A training set (Supplementary Fig. S4). Score thresholds in the algorithm were selected to achieve greatest classification accuracy in the training set. This algorithm yielded accuracy 0.83 95% CI 0.81–0.85 and Kappa 0.79 in Cohort A validation set. The important Stem-like subset was predicted with accuracy 0.92 95% CI 0.91–0.94, Kappa = 0.79.

### Identification of samples with mixed subtypes using ColoType subtyping scores

Each of the four ColoType subtyping scores was defined as an independent predictor of the corresponding CMS subtype. The classification threshold for CMS1-score, e.g., was defined so that only samples with CMS1-score above the threshold, subsequently denoted CMS1+, could reasonably be classified into CMS1. Let CMS1- denote the samples with CMS1-score below the classification threshold, and similarly for other subtypes. We consider a tumor to have *mixed type* if it is positive for multiple CMS scores. We found that 21% of Cohort A were of mixed type, while 66% were positive for a single type and 13% were unclassified. The distributions of mixed types in Cohort A (Supplementary Table S4) showed that samples with mixed type CMS2+ and CMS4+ were most frequent (8.8%), followed by CMS1+ and CMS4+ (6.6%), CMS1+ and CMS3+ (3.5%), CMS2+ and CMS3+ (1.5%), and others containing fewer than 1% of samples. Note that almost no samples were in CMS1+ and CMS2+ or CMS3+ and CMS4+.

Samples of mixed types may give insight into discordant classifications by multiple classifiers. For samples that were positive for a single type in the Cohort A validation set, ColoType predicted the CRCSC CMS subtype with accuracy 0.96 95% CI 0.95–0.98, Kappa = 0.95. In contrast, for samples of mixed types, the accuracy was only 0.72 (95% CI 0.65–0.77), Kappa = 0.58. Apparently, a sample of mixed type exhibits features of both positive subtypes, and different classifiers may reasonably predict either subtype. Reporting the continuous score values for all subtypes is a more complete description of the tumor.

## Discussion

Patient treatment for colorectal cancer today is largely determined by tumor stage. To enable better outcomes, patients need to be stratified in a manner that takes into account the molecular heterogeneity of the disease. The consensus molecular subtypes effectively stratify CRC tumors by tumor biology, however the lack of a commercial assay for subtyping has made clinical applications impossible. As a step towards filling this gap, we developed the 40-gene ColoType assay for CMS subtyping that can be implemented with targeted RNA-sequencing of FFPE tissue.

We showed (Supplementary Fig. S3, Fig. 3) that when using size factor normalized expression values, ColoType, CMScaller and CMSclassifier produced highly correlated subtypes in multiple datasets, independent of whole-genome RNA-seq library preparation [selection by poly(A) tail or rRNA depletion] or tissue source (frozen or FFPE). In addition, the subtypes produced by these systems largely agreed with the gold-standard ones in TCGA-COAD (Cohort C). These results show that whole-genome RNA-seq can be the basis for studying CMS subtypes using frozen or FFPE tissues if size factor normalization is used to measure gene expression. Moreover, the CMS subtypes produced by whole-genome sequencing had significant agreement with those obtained with ColoType by targeted RNA-seq in the Marshfield cohort, supporting ColoType and RNA-seq as bases for CMS subtyping in both research and clinical settings.

Targeted RNA-seq is increasingly being used for clinical diagnostics^25,35,36^. For solutions requiring, say 25 to a few hundred genes, the cost of a sample’s library preparation is at least comparable to the cost of materials for other technologies. Moreover, when targeting comparatively few genes (< 100), it is possible to sequence many samples in one run, allowing for overall cost-effective sample analysis, with turn-around times that meet clinical expectations. The increasing prevalence of next-generation sequencing instrumentation in clinical pathology laboratories further supports the choice of this technology for a commercial clinical solution.

Research following the derivation of the CMS subtypes suggested that classification of a tumor into a single subtype may be inappropriate due to intratumoral heterogeneity^37–40^. Rather than classifying a tumor into a single subtype, it may be more accurate to measure the concentration of a particular cell type throughout a tumor. To this end, ColoType was designed to report a continuous measurement of the prevalence of each CMS subtype using mRNA harvested throughout the tumor sample. ColoType reports a single subtype that has greatest prevalence, when such exists, but we envision that ColoType will report continuous score values for all subtypes for completeness.

Significant intratumoral heterogeneity may be a source of samples with mixed types. One instance of heterogeneity is the observation that cells of a mesenchymal type (CMS4) are more often found at the invasive front than the core of the tumor^39^. This is consistent with the observation that mixed types involving CMS4+ were most common in Cohort A. Also note that CMS4 subtypes do not appear to occur as precancerous lesions^41^. Additional research is required to analyze the degree to which tumors of mixed type have biological features characteristic of the component subtypes, and how they arise in the span of a patient’s disease.

Clinical application of ColoType will require establishing analytic validity of a targeted RNA-seq implementation in a CLIA certified pathology laboratory. Additional studies are also needed to verify the clinical utility of ColoType. The ability of CMS subtyping to predict response to specific drugs has been reported, however subtypes were defined using in-house solutions in some of these studies. These results need to be calibrated to the ColoType scores for optimal clinical application. It is possible that the greatest predictive significance results from score thresholds other than the classification thresholds; in other words, the continuous scores may be more significantly predictive of drug response than the subtypes themselves.

In summary, we developed the 40-gene ColoType signature for CMS subtyping CRC tumors using whole-genome assays or targeted RNA-seq of frozen or FFPE tissues. The resulting subtypes correlated highly with those reported by multiple independent subtype systems. Since some CRC tumors exhibit intratumoral heterogeneity, ColoType’s emphasis on continuous scores likely provides a more accurate description of tumor biology than classification into a single subtype. The planned analytic validation of the ColoType targeted RNA-seq assay will enable accurate and reproducible CMS subtype analysis for clinical applications.

## Supplementary information

Supplementary Information.

Supplementary Table 1.

Supplementary Table 2.

Supplementary Table 3.

Supplementary Table 4.

## Data Availability

Gene expression values for samples in Cohorts A, B and C can be obtained as described in Methods. ColoType score values and classifications for samples in Cohorts A, B and C are available in Supplementary Table S2. Whole-genome RNA-sequencing data for samples in the Marshfield Cohort are available through NCBI Gene Expression Omnibus as accession number GSE152430.
