# Supplementary material for: ColoType: a forty gene signature for consensus molecular subtyping of colorectal cancer tumors using whole-genome assay or targeted RNA-sequencing
Source: Sci Rep. 2020 Jul 21;10:12123. doi: 10.1038/s41598-020-69083-y (PMC7374173; doi:10.1038/s41598-020-69083-y)
Supplement: Supplementary file 1 — Supplementary Information. [file 41598_2020_69083_MOESM1_ESM.pdf]

ColoType: A forty gene signature for consensus molecular subtyping of colorectal cancer tumors using whole-genome assay or targeted RNA-sequencing

Steven A Buechler, Melissa T Stephens, Amanda B Hummon, Katelyn Ludwig, Emily Cannon, Tonia C Carter, Jeffrey Resnick, Yesim Gökmen-Polar, Sunil S Badve

SUPPLEMENTARY METHODS

S1 Multigene risk score classification (MRS) method

Following is a more detailed description of the multigene risk score classification method introduced in Methods.

S1.1 Gene risk scores for classification

Gaussian mixture models (GMM) have long been used for supervised classification[1-4], and can be applied in our setting as follows. To predict membership in a subtype *X* of a population **P** using a gene *g*, we can compute a GMM of the vector *v* of expression values of *g* in **P**, identify the component of the GMM that is enriched with members of *X*, and predict that all members of the component are in *X*. The simplest form of a GMM is when it defines two connected components, in other words, the samples with expression below a threshold (low component) and those with expression above a threshold (high component). Numerically, this prediction could be represented by assigning 1 to each sample in the enriched component and 0 to all other samples. In this form, *v* has been replaced by a binary variable, with a GMM determining the threshold separating samples with values 0 from samples with values 1; samples with value 1 are predicted to be in *X*.

One disadvantage of a GMM prediction model is that in a larger set of samples, the threshold defining components in the original population may not exactly match the threshold between components of a GMM in the larger population. Bootstrap methods have previously been used to assess the uncertainty in how the parameters of a GMM generalize[5-7]. Here, bootstrap resampling is used to measure the uncertainty in the threshold between two components in a mixture model as follows. Given expression values *v* of a gene *g* in **P** and GMM of *v* with two components as above, the risk score *r* for *g* is defined so that for each sample *x* in **P**, the value of *r* at *x* is the fraction of bootstrap samples B_j, $j = 1,...,100$ of **P** containing *x* in which *x* is in the high component in a two-component GMM of the restriction of *v* to B_j. Note that in this way, the risk score of *g* is a measure of the probability that a sample is in the high component of a GMM computed in some larger population containing **P**. When a GMM for a gene *g* has more than two components, or has a component composed of multiple disjoint intervals, a risk score can be defined for any boundary point between components following merging intervals above the boundary into a high component, and those below the boundary into a low component, and repeating the above method for two connected components. On the other

hand, a gene may not give rise to any risk scores when its expression is approximately normally distributed in **P**. When using a risk score r to predict membership in a subset it is most convenient when high values of r are more likely to be in the subset. In a setting in which low expression values of the gene are more likely to be in the subset, r can be replaced by $1 - r$ in a predictive model.

One measure of the distribution of a risk score is the fraction of values greater than 0.5, termed the *fraction-high*. A risk score is called *nondegenerate* if its fraction-high is between 0.1 and 0.9. For a given gene g, a nondegenerate risk score r for g in a cohort **M** is called equivalent to a nondegenerate risk score s for g in a cohort **N**, if the absolute value of the difference of fraction-high values for r and s is < 0.10. When attempting to match a risk score r with one in another cohort, if multiple equivalent risk scores are available, we select the one with fraction-high closest to the fraction-high of r. In this study, only nondegenerate risk scores were considered.

S1.2 MRS score for subtype prediction in a training set

Above, we described how a risk score of a gene can represent a single-gene predictor of a subtype X in a cohort **P**. Here, given a panel of genes $g_1,\ldots,g_n$ and risk scores $r_1,\ldots,r_n$, each of which is a predictor of X, we describe how to define a *Multigene Risk Score* (MRS score) for prediction of X that models a consensus prediction of all of the genes.

In an MRS classification system for a subtype X, a panel of genes $g_1,\ldots,g_n$ and risk scores $r_1,\ldots,r_n$ is first selected in a training set. To define a single continuous score to predict membership in X, first compute r, the vector of means of $r_1,\ldots,r_n$; i.e., the value of r at any sample in the population is the mean of the values of $r_1,\ldots,r_n$ at the given sample. Since membership in X is known in this training cohort, we can define a score S_0 so that the value of S_0 for a sample x, is the positive predictive value of $r(x)$; i.e. $S_0(x)$ is the ratio of the number of samples in X whose r-value is $\geq r(x)$ divided by the total number of samples whose r-value is $\geq r(x)$. Then, an *MRS score S* is obtained by applying loess smoothing to S_0 with the constraint that S is smooth and non-decreasing with respect to values of r.

An MRS predictive score S defines a discrete predictor of membership in X by selecting a threshold c so that samples with value of $S > c$ are predicted to be in X, and those with value of $S \leq c$ are predicted to not be in X. In this way each possible threshold is associated with a sensitivity, specificity and Youden index (sensitivity + specificity – 1) in the training cohort. As the *classification threshold* for S, we select the score at which the Youden index is maximal[8] in the training cohort.

S1.3 MRS classification of a system of subtypes

Given a system of disjoint subtypes $X_1,\ldots,X_m$, each associated with an MRS predictive score, we classify a sample into one of the subtypes using the MRS scores as discriminants. Specifically, we predict a sample to be in that subtype whose score is above the classification threshold and maximal among such scores, unless none of the scores is above the classification threshold, in which case the sample is unclassified.

S1.4 MRS score for subtype prediction using a reference set

Once an MRS subtyping score has been defined for classifying a subtype in a training cohort **P***,* it can be extended to a new cohort of patients **Q** using results from **P** as a reference set. First, for $r_1,\ldots,r_n$ risk scores for the selected panel genes in **P**, identify (if possible) risk scores $s_1,\ldots,s_n$ for the panel genes in **Q** so that each s_i is equivalent to r_i. Let Y be the vector of means of $r_1,\ldots,r_n$ in **P**, Z the vector of means of $s_1,\ldots,s_n$ in **Q**. Let *S* be the MRS score in **P** derived from Y as in S1.2. We define an MRS subtyping score *T* in **Q** using interpolation. In detail, for u a sample in the new cohort **Q**, let z = Z(u), identify a sample t in **P** whose value of Y is closest to z, and define *T*(u) to be *S*(t).

S1.5 MRS single sample predictor using a reference set

The use of an MRS classification system in a clinical setting requires that it can be applied to a single new sample. This can be accomplished by the following interpolation when the expression measurements for the genes underlying the system are normalized to the expression measurements in the reference set **P**. Let u be the new sample, and *S* an MRS predictive score with underlying risk scores $r_1,\ldots,r_n$. Let *x* be the vector of expression values of gene g_i from which risk score r_i was derived. Let t_i be the sample in **P** with expression value of g_i closest to the expression level of g_i for u. Then, associate with u, the risk score value of r_i at t_i. Doing so for each of *n* panel genes computes the required risk score values for u that enable computation of the MRS score as in S1.4.

References

1. Fraley, C. & Raftery, A. E. Model-Based Clustering, Discriminant Analysis, and Density Estimation. *Journal of the American Statistical Association* **97**, 611-631 (2002).

2. Scrucca, L., Fop, M., Murphy, T.,Brendan & Raftery, A.,E. mclust 5: Clustering, Classification and Density Estimation Using Gaussian Finite Mixture Models. *The R Journal* **8**, 289 (2016).

3. Prabakaran, I. *et al*. Gaussian Mixture Models for Probabilistic Classification of Breast Cancer. *Cancer Res* **79**, 3492-3502 (2019).

4. McNicholas, P. D. in *Mixture model-based classification,* xiii-212 (CRC Press, Taylor & Francis Group, Boca Raton, FL, 2017).

5. McLachlan, G. J. On bootstrapping the likelihood ratio test statistic for the number of components in a normal mixture. *Appl. Stat.* **36**, 318-324 (1987).

6. Newton, M. A. and Raftery, A. E. Approximate Bayesian inference with the weighted likelihood bootstrap (with discussion). *Journal of the Royal Statistical Society: Series B (Statistical Methodology)* **56**, 3-48 (1994).

7. Jaki, T., Su, T., Kim, M. & Lee Van Horn, M. An evaluation of the bootstrap for model validation in mixture models. *Commun Stat Simul Comput* **47**, 1028-1038 (2018).

8. Le, C. T. A solution for the most basic optimization problem associated with an ROC curve. *Stat Methods Med Res* **15**, 571-584 (2006).

SUPPLEMENTARY FIGURES

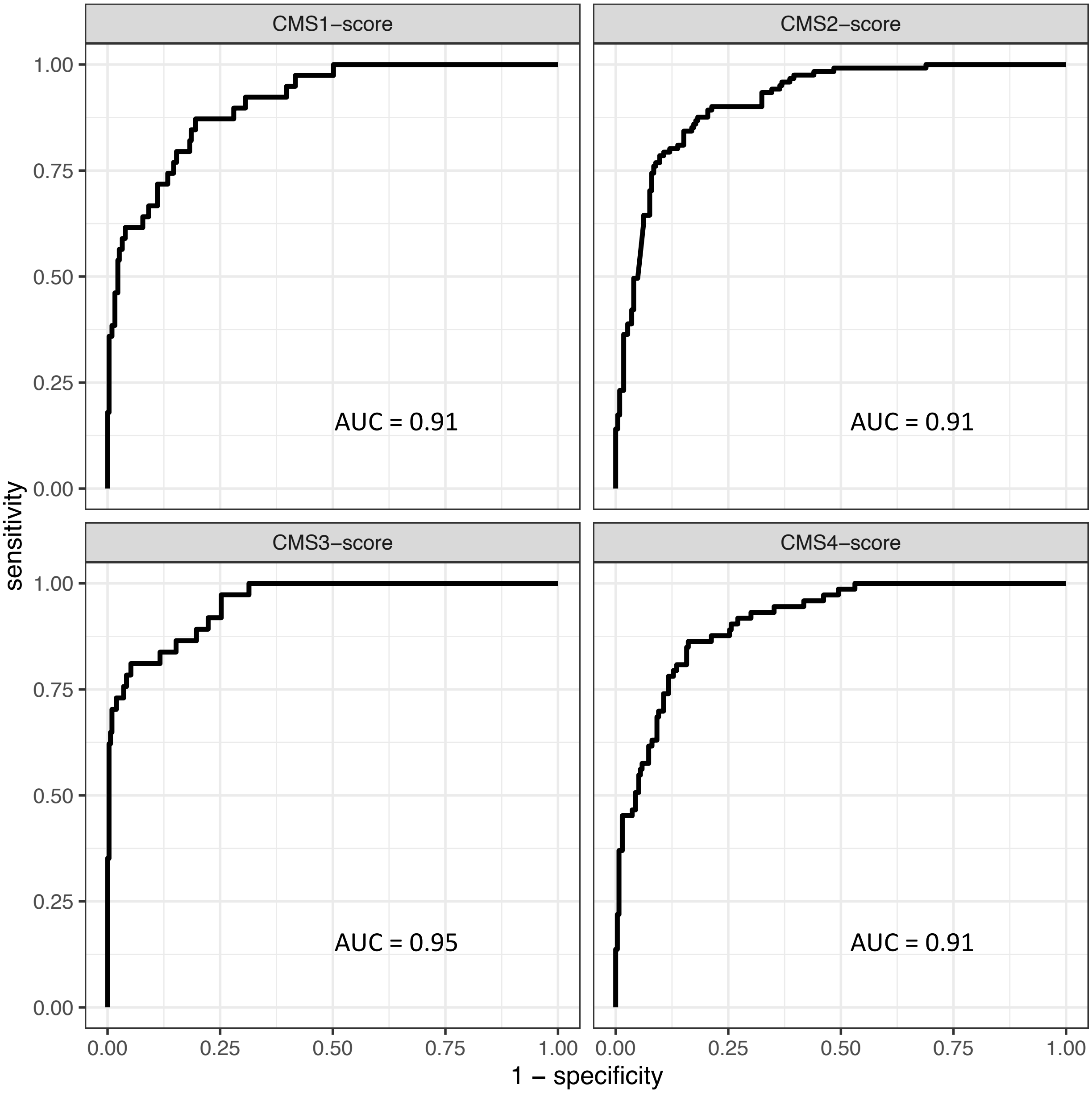

Supplementary Figure S1. Receiver operator characteristic (ROC) curves were plotted for ColoType CMS1-score, CMS2-score, CMS3-score, and CMS4-score, for samples in the Cohort B (PETACC-3) validation set (n = 346). Area under the curve (AUC) values are displayed on the panels.

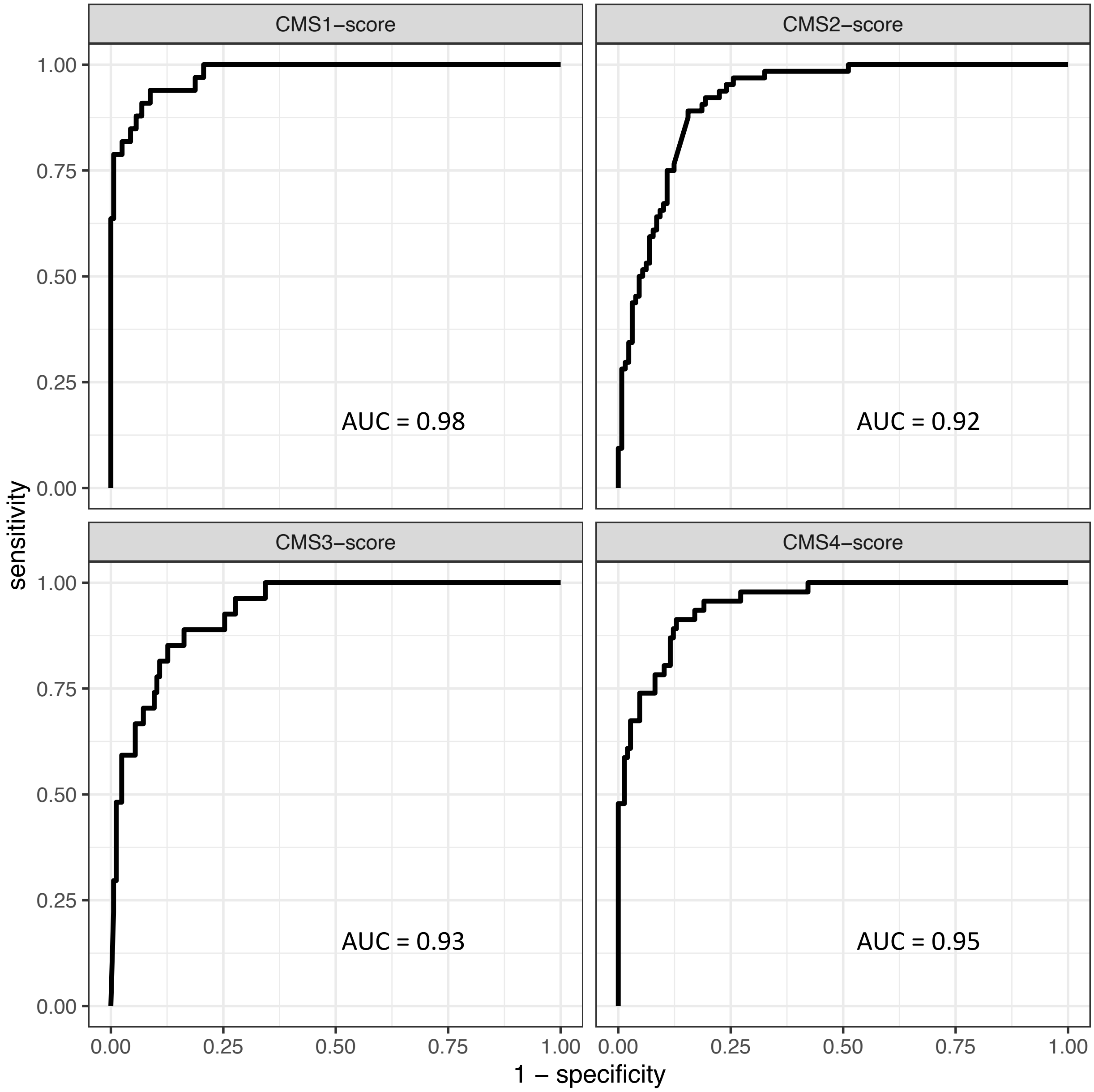

Supplementary Figure S2. Receiver operator characteristic (ROC) curves were plotted for ColoType CMS1-score, CMS2-score, CMS3-score, and CMS4-score, for samples in the Cohort C (TCGA-COAD) validation set (n = 193). Area under the curve (AUC) values are displayed on the panels.

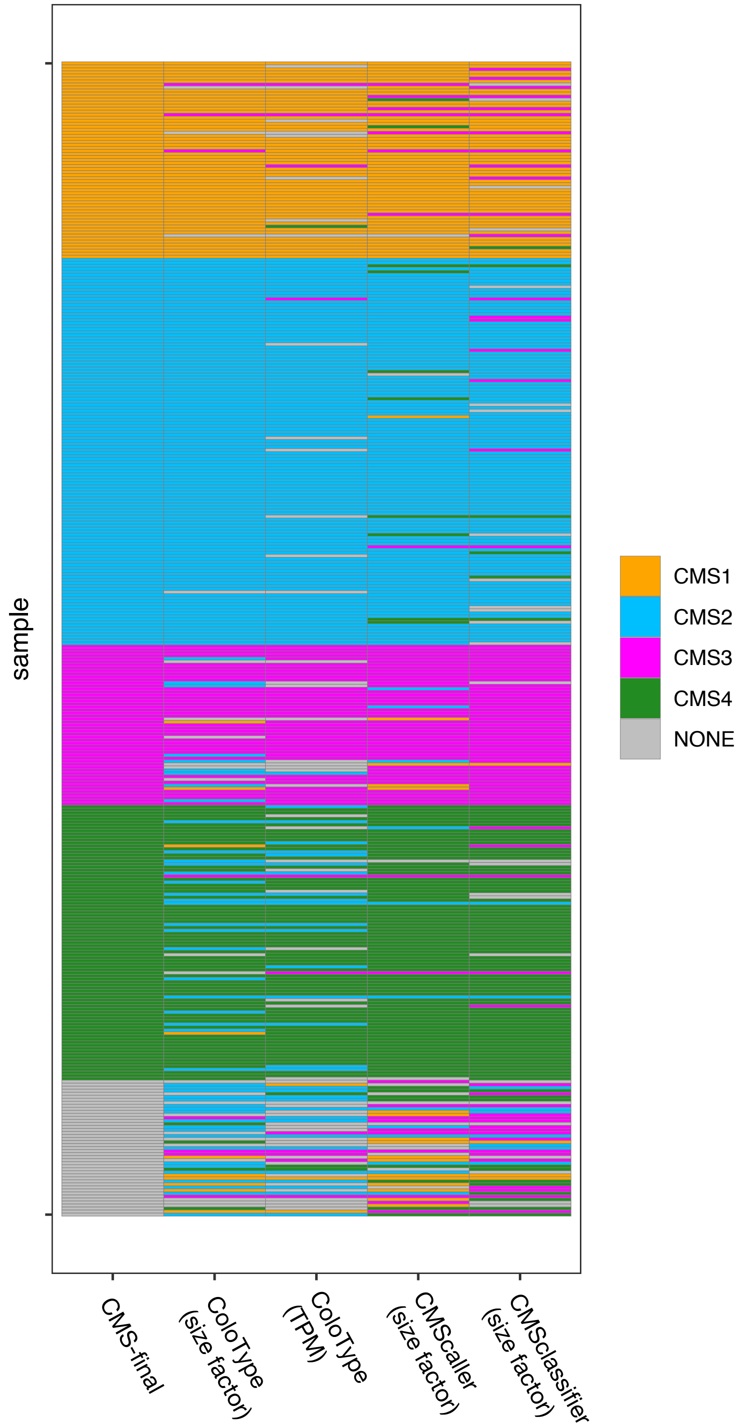

Supplementary Figure S3. For each sample in COAD, the CMS classification is displayed for the classification methods: CRCSC classification (CMS-final), ColoType with size factor normalization, ColoType with TPM normalization, and CMScaller and CMSclassifier, both using size factor normalized expression values.

ColoType CMS	Condition	Prediction
CMS2	Enterocyte-score > 0.76	Enterocyte
CMS2	Enterocyte-score ≤ 0.76 & CMS4-score ≤ 0.51	TA
CMS2	Enterocyte-score ≤ 0.76 & CMS4-score > 0.51	Stem-like
not CMS2	Enterocyte-score > 0.84	Enterocyte
CMS1	Enterocyte-score ≤ 0.84	Inflammatory
CMS3	Enterocyte-score ≤ 0.84	Goblet-like
CMS4	Enterocyte-score ≤ 0.84	Stem-like
NONE	Enterocyte-score ≤ 0.84	NONE

Supplementary Figure S4. Rules for a CRCassigner subtype classifier based on the ColoType subtypes, the ColoType CMS4-score, and the newly defined Enterocyte-score, based on expression of *CA1* and *CA2*. The score thresholds used were identified in the Cohort A training set by maximizing prediction accuracy.
